# Usefulness of Next-Generation Sequencing in Excluding Bovine Leukemia Virus as a Cause of Adult Camel Leukosis in Dromedaries

**DOI:** 10.3390/pathogens12080995

**Published:** 2023-07-29

**Authors:** Ulrich Wernery, Jade L. L. Teng, Yuanchao Ma, Joerg Kinne, Man-Lung Yeung, Safna Anas, Susanna K. P. Lau, Patrick C. Y. Woo

**Affiliations:** 1 Central Veterinary Research Laboratory, Dubai, United Arab Emirates; jkinne@cvrl.ae (J.K.);; 2 Faculty of Dentistry, The University of Hong Kong, Hong Kong Special Administrative Region, China; llteng@hku.hk; 3 Department of Microbiology, School of Clinical Medicine, Li Ka Shing Faculty of Medicine, The University of Hong Kong, Hong Kong Special Administrative Region, China; myc0102@connect.hku.hk (Y.M.); pmlyeung@hku.hk (M.-L.Y.); skplau@hku.hk (S.K.P.L.); 4 State Key Laboratory of Emerging Infectious Diseases, Li Ka Shing Faculty of Medicine, The University of Hong Kong, Hong Kong Special Administrative Region, China; 5 Department of Clinical Microbiology and Infection Control, The University of Hong Kong-Shenzhen Hospital, Shenzhen 518053, China; 6 Carol Yu Centre for Infection, Li Ka Shing Faculty of Medicine, The University of Hong Kong, Hong Kong Special Administrative Region, China; 7 Centre for Virology, Vaccinology and Therapeutics, Hong Kong Science and Technology Park, Hong Kong Special Administrative Region, China; 8 Doctoral Program in Translational Medicine and Department of Life Sciences, National Chung Hsing University, Taichung 402, Taiwan; 9 The iEGG and Animal Biotechnology Research Center, National Chung Hsing University, Taichung 402, Taiwan

## Abstract

Adult camel leukosis is an emerging hematological and neoplastic disease in dromedaries. It has been hypothesized that bovine leukemia virus (BLV) or its genetic variants may be associated with adult camel leukosis. In this study, we used next-generation sequencing (NGS) to detect all possible viruses in five lung samples from five dromedaries with histopathological evidence of adult camel leukosis and four tissue samples from two control dromedaries. A total throughput of 114.7 Gb was achieved, with an average of 12.7 Gb/sample. For each sample, all the pair-end 151-bp reads were filtered to remove rRNA sequences, bacterial genomes and redundant sequences, resulting in 1–7 Gb clean reads, of which <3% matched to viruses. The largest portion of these viral sequences was composed of bacterial phages. About 100–300 reads in each sample matched “multiple sclerosis-associated retrovirus”, but manual analysis showed that they were only repetitive sequences commonly present in mammalian genomes. All viral reads were also extracted for analysis, confirming that no BLV or its genetic variants or any other virus was detected in the nine tissue samples. NGS is not only useful for detecting microorganisms associated with infectious diseases, but also important for excluding an infective cause in scenarios where such a possibility is suspected.

Adult camel leukosis is a hematological and neoplastic disease in adult dromedaries characterized by extensive lymphocytic infiltration of pulmonary tissue and lymph nodes. From 1988 to 2004, among 850 necropsies on adult dromedaries performed in our Central Veterinary Research Laboratory in Dubai, the United Arab Emirates, 12 (1.4%) were diagnosed with adult camel leukosis [1]. In the following nine years (2005 to 2013), 12 additional cases (1.56%) of adult camel leukosis out of 680 necropsies on adult dromedaries were diagnosed (unpublished data). Notably, in the last eight years (2014 to 2022) out of 1153 necropsies on adult dromedaries performed in our laboratory in Dubai, 77 (6.67%) were confirmed to have adult camel leukosis, representing a more than 300% increase in incidence (unpublished data). 

Bovine leukemia virus (BLV) is a deltaretrovirus which is able to infect B-lymphocytes in cattle and induces proliferation in these cells, which subsequently leads to enzootic bovine leukosis (EBL) in cattle—a malignant neoplastic disease that mainly involve the lymph nodes, in the form of lymphoma or lymphosarcoma [2]. Since BLV has been shown to be transmitted to sheep, buffalos and other small ruminants, causing an EBL-like disease in these animals [3,4,5], and there are a lot of morphological similarities between EBL and adult camel leukosis, there has been a lot of interest in seeing whether this virus can also lead to adult camel leukosis in dromedaries. Although preliminary studies using PCR amplification for BLV on blood samples of apparently healthy dromedaries have not shown any positive results [6,7], there is still concern as to whether BLV may be associated with adult camel leukosis in dromedaries, as PCR amplification may not be able to pick up BLV or its genetic variants with significant differences from BLV, and the virus may be present in the tissue of dromedaries with adult camel leukosis instead of blood in healthy camels. To answer this question, we used next-generation sequencing (NGS) to detect all possible viruses in tissue samples of adult dromedaries with adult camel leukosis. 

Five lung tissue samples from five dromedaries with histopathological evidence of adult camel leukosis (Figure 1) and four tissue samples [lung (*n* = 1), liver (*n* = 1), spleen (*n* = 1) and lymph node (*n* = 1)] from two control dromedaries with other diagnosis were collected by the Central Veterinary Research Laboratory in Dubai, the United Arab Emirates. Total RNA was extracted from the tissue samples using RNeasy kit (QIAGEN Inc., Germantown, MD, USA). After reverse transcription and random PCR, amplicons of <400 bp were removed using AMPure XP (Beckman Coulter Life Sciences, Loveland, CO, USA) beads according to the manufacturer’s instructions. One nanogram of random purified PCR product was used for the subsequent steps of libraries preparation based on the protocol of the Nextera XT DNA Sample Prep Kit (Illumina, San Diego, CA, USA) with IDT UDI 10 nt Nextera primer pairs. Briefly, DNA fragments with adaptors were generated via tagmentation reaction at 55 °C for 5 min. Adaptor-ligated libraries were generated in a 50 μL reaction volume with 12 cycles of polymerase chain PCR. The enriched libraries were validated by Agilent Bioanalzyer, Qubit and qPCR for quality control analysis. The libraries were denatured and diluted to optimal concentration. Illumina NovaSeq 6000 was used for pair-end 151 bp sequencing. 

A total throughput of 114.7 Gb was achieved. Using software from Illumina (bcl2fastq), sequencing reads were assigned to individual samples, giving rise to an average throughput of 12.7 Gb per sample. For sequence quality, an average of 88% of the bases achieved a quality score of Q30, where Q30 denotes the accuracy of a base call to be 99.9% (Table 1). For each sample, all the pair-end 151-bp reads were filtered using deconseq 0.4.3 to remove ribosomal RNA sequences and bacterial genomes. Redundant sequences were removed using cd-hit 4.8.1, resulting in 1–7 Gb clean reads which were subjected to downstream sequence analysis. Of these clean reads, >97% matched with host genomes, whereas <3% matched with viruses. The largest portion of these viral sequences was assigned to bacterial phages. About 100–300 reads in each sample matched with multiple sclerosis-associated retrovirus. These “retrovirus” reads were extracted for assembly and further analysis. Manual analysis showed that these are repetitive sequences that are commonly present in mammalian genomes, and this has led to false matches with multiple sclerosis-associated retrovirus [8]. All viral reads were also extracted for analysis, confirming that no BLV or its genetic variants or any other virus reads were detected in the nine tissue samples collected from dromedaries with adult camel leukosis or other diagnoses.

In this study, we confirmed that BLV infection is not associated with adult camel leukosis in dromedaries using NGS. As BLV is able to infect sheep, buffalos and other small ruminants in addition to cattle, it is of interest to see if this retrovirus can also infect dromedaries. In 2015 and 2018, two studies from Iran and Algeria used nested PCR for the *env* gene and PCR for the *pol* gene, respectively, to detect BLV from 122 and 111 blood samples of asymptomatic dromedaries, revealing negative results [6,7]. In addition, another serological study in 2020 also showed that there were no BLV antibodies in 100 serum samples from dromedaries in Egypt using IDEXX Leukosis Serum Screening ELISA [9]. In the present study, we used NGS to confirm the absence of BLV in the lung, liver, spleen and lymph node of histopathologically proven cases of adult camel leukosis from Dubai. This is in line with the previous molecular and serological tests on blood samples in healthy dromedaries from other parts of the Middle East and North Africa [6,7,9]. 

NGS is not only useful for detecting microorganisms in human or animals with infectious diseases, it is also important for excluding an infective cause in clinical scenarios in which such a possibility is suspected. When NGS technologies first appeared in the market, they were mainly used for genome sequencing. With the advancement of sequencing chemistries and computational capacity, NGS technologies have matured into clinical applications in recent years. In the clinical setting for human infections, NGS is used most often for patients who have a fever without localizing features, or for culture-negative infections. For example, we have recently reported its application in confirming the first case of listeria meningitis in a patient with an autoantibody against interferon gamma, as well as understanding the spectrum of Q fever, fungal infections and culture-negative meningitis and encephalitis [10,11,12,13]. As for animals, the application of NGS to veterinary testing has been reported in the literature, ranging from shotgun metagenomics for novel/known pathogen detection to amplicon sequencing for targeted detection of multiple pathogens in animal samples [14,15,16]. This technology may allow us to discover some previously undescribed animal pathogens with global importance. For example, Aghazadeh et al. discovered a novel domestic cat hepadnavirus in an immunocompromised domestic cat via NGS [17]. Since its first discovery in Australia in 2018, this virus has been found to be globally distributed in domestic cats, with cases reported in Hong Kong [18], Malaysia [19], the United Kingdom [20], the United States [21] and Japan [22]. In addition to its contribution in confirming the etiology of an infection, NGS is also crucial in excluding a microbiological cause for clinical settings in which an infective cause is a possibility. For example, in our recent study on patients with suspected meningitis and encephalitis, NGS was useful for excluding an infective cause in two patients who were subsequently diagnosed with autoimmune encephalitis, and corticosteroid was hence confidently used for treatment [12]. In the present study, NGS has also conclusively excluded BLV or its genetic variants as a cause of adult camel leukosis. A similar approach can be used for other animal diseases in which an infective cause is a possibility. 

Currently, the main hurdle for the widespread use of NGS in veterinary diagnostic laboratories is the high cost. In the past, NGS required expensive equipment such as Illumina’ s HiSeq System, Life Technologies’ SOLiD System, and PacBio’ s RS/Sequel Systems, which could cost up to USD 10 million, including machine costs and necessary infrastructure. However, newer models of sequencers such as Illumina’ s MiSeq System and Life Technologies’ PGM System are now available, and these are less expensive and deliver high-throughput sequencing on the bench, using less laboratory space. To reduce costs, it is more economical to sequence multiple specimens in a single run, as the sequencing data generated by each platform are generally higher in quantities than required per specimen. This strategy reduces the cost of sequencing one sample to around USD 500. In 2016, Oxford Nanopore Technologies introduced its first sequencing device, the MinION, which is a portable and affordable alternative to conventional NGS sequencers. It costs around USD 1,000. However, sequencing one sample using the MinION device still costs around USD 1,500 (machine cost inclusive), which is still too expensive for most veterinary practices. Veterinary diagnostics services for NGS-based pathogen detection have emerged in recent years. For example, the cost of testing individual samples using this NGS technology to detect over 20 different pathogens is around USD 200 [23], making it a more favorable option for some veterinary practices. When the cost of NGS continues to decrease and expertise becomes more readily available, NGS could become a valuable tool for routine diagnosis of animal infections.

## Figures and Tables

**Figure 1:**
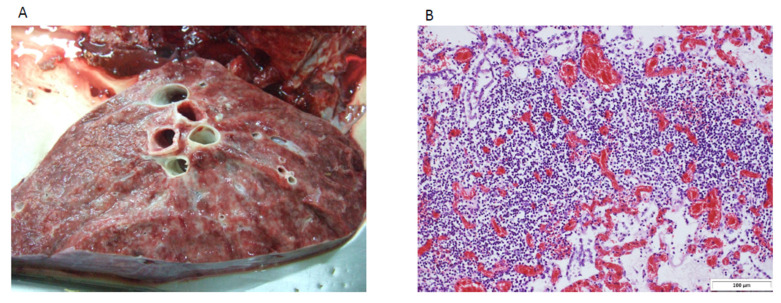
Necropsy samples of a dromedary with adult camel leukosis. (**A**) Lung tissue showed diffuse grayish consolidation due to extensive lymphatic infiltration. (**B**) Histological examination of the lung tissue using hematoxylin–eosin staining showed extensive lymphatic infiltration.

**Table 1:** Read data from high-throughput sequence analyses of individual samples from dromedaries.

Samples	Number of Raw Reads (Read 1 + Read 2)	Total Throughput (Gb)	Percentage of Bases with Quality Core ≥30
Dromedaries with leukosis			
Lung tissue of dromedary 1 (D1607/2021)	71,921,466	10.9	86
Lung tissue of dromedary 2 (D2441/2021)	71,753,206	10.8	88
Lung tissue of dromedary 3 (D2833/2021)	82,897,096	12.5	88
Lung tissue of dromedary 4 (D2868/2021)	87,851,042	13.3	87
Lung tissue of dromedary 5 (D3219/2021)	77,300,200	11.7	89
Control dromedaries with other diagnosis			
Spleen tissue of dromedary 6 (D135/2020)	87,717,290	13.2	88
Lymph node of dromedary 6 (D135/2020)	90,616,970	13.7	88
Liver tissue of dromedary 7 (D3321/2021)	89,520,788	13.5	88
Lung tissue of dromedary 7 (D3321/2021)	99,676,894	15.1	89

## Data Availability

The raw datasets presented in this study were deposited in the U.S. National Center for Biotechnology Information’s Sequence Read Archive as bioproject number PRJNA984175.
